# The effect of duloxetine on mechanistic pain profiles, cognitive factors and clinical pain in patients with painful knee osteoarthritis—A randomized, double‐blind, placebo‐controlled, crossover study

**DOI:** 10.1002/ejp.1988

**Published:** 2022-06-13

**Authors:** Kristian Kjær‐Staal Petersen, Asbjørn Mohr Drewes, Anne Estrup Olesen, Nadia Ammitzbøll, Davide Bertoli, Christina Brock, Lars Arendt‐Nielsen

**Affiliations:** ^1^ Center for Neuroplasticity and Pain, Department of Health Science and Technology, Faculty of Medicine Aalborg University Aalborg Denmark; ^2^ Center for Mathematical Modeling of Knee Osteoarthritis (MathKOA), Department of Material and Production, Faculty of Engineering and Science Aalborg University Aalborg Denmark; ^3^ Department of Clinical Medicine Aalborg University Aalborg Denmark; ^4^ Mech‐Sense, Department of Gastroenterology and Hepatology Aalborg University Hospital Aalborg Denmark; ^5^ Department of Clinical Pharmacology Aalborg University Hospital Aalborg Denmark

## Abstract

**Background:**

Duloxetine is indicated in the management of pain in osteoarthritis. Evidence suggests that duloxetine modulates central pain mechanisms and cognitive factors, and these factors are assumed contributing to the analgesic effect. This proof‐of‐mechanism, randomized, placebo‐controlled, crossover, double‐blinded trial evaluated the effect of duloxetine on quantitative sensory testing (QST), cognitive factors and clinical pain in patients with osteoarthritis and to predict the analgesic effect.

**Methods:**

Twenty‐five patients completed this cross‐over study with either 18‐week duloxetine (maximum 60 mg/daily) followed by placebo or vice‐versa. Pressure pain thresholds, temporal summation of pain and conditioned pain modulation were assessed using cuff algometry. The Hospital Anxiety and Depression Scale and the Pain Catastrophizing Scale evaluated cognitive factors. Clinical pain was assessed using Brief Pain Inventory and Western Ontario and McMaster Universities Osteoarthritis Index. Linear regression models were used to predict the analgesic effect of duloxetine.

**Results:**

Depending on the clinical pain outcome, 40%–68% of patients were classified as responders to duloxetine. Linear regression models predicted the analgesic effect (predictive value of 45%–75% depending on clinical pain outcome parameter) using a combination of pretreatment QST parameters, cognitive factors and clinical pain. No significant changes were found for QST, cognitive factors or clinical pain on a group level when comparing duloxetine to placebo.

**Conclusion:**

A combination of pretreatment QST, cognitive factors and clinical pain was able to predict the analgesic response of duloxetine. However, in this relatively small study, duloxetine did not selectively modulate QST, cognitive factors or clinical pain intensity when compared with placebo.

**Significance:**

Duloxetine is proposed as a treatment for chronic pain. Pre‐clinical trials suggest that duloxetine provides analgesia through modulation of descending pain inhibitory pathways or through improvements in cognitive factors. The current study demonstrates that pretreatment mechanistic pain profiling, cognitive factors and clinical pain can predict the analgesic effect of duloxetine and that only a subset of patients might benefit from duloxetine treatment.

## 1 INTRODUCTION

Osteoarthritis is prevalent in both high‐ and low‐income countries (Safiri et al., 2020). The prevalence of OA increases with life style changes (Reyes et al., 2016) and age (Berenbaum et al., 2018), and the global prevalence of OA is expected to continue its rise.

Pain is the hallmark symptom of OA and the 2019 Osteoarthritis Research Society International guidelines included duloxetine (a serotonin–noradrenalin reuptake inhibitor antidepressant) for the treatment of patients with OA and widespread pain and/or depression (Bannuru et al., 2019). Seven randomized controlled trials have demonstrated analgesic effects of duloxetine when compared to placebo in (Blikman et al., 2022; Chen et al., 2021) although the mechanism‐of‐action in pain is not completely elucidated.

Recent evidence suggests that OA clinical pain is increased by cognitive factors such as depression, anxiety and pain catastrophizing (Edwards et al., 2011; Larsen, Laursen, Simonsen, et al., 2021). As an antidepressant, for example, duloxetine, improves symptoms of depression and anxiety in patients with OA (Cipriani et al., 2018; Lunn et al., 2015). Therefore, it is likely that a potential analgesic effect of duloxetine could be partly mediated through these factors.

Quantitative sensory testing (QST), for example, pressure pain thresholds (PPTs), temporal summation of pain (TSP) and conditioned pain modulation (CPM) have been used to assess pain sensitivity in patients with OA (Arendt‐Nielsen et al., 2015, 2016; Petersen, 2021; Petersen et al., 2016; Petersen, Olesen, et al., 2019; Petersen, Simonsen, et al., 2019). Accumulating evidence suggests that severe OA is associated with lower PPTs, facilitated TSP and impaired CPM (Arendt‐Nielsen et al., 2015) and that these mechanistic pain biomarkers might hold predictive value for pain management with different therapies (Edwards et al., 2016; Kjær et al., 2019; Petersen et al., 2015, 2016, 2021; Petersen, Simonsen, et al., 2019). Pre‐clinical studies have shown that serotonin and noradrenaline are important neurotransmitters for the descending pain inhibitory pathways (Bannister et al., 2017; Lockwood et al., 2019), hence suggesting that the human proxy for assessing the descending modulation CPM (Bannister & Dickenson, 2017) may be useful to study such pain modulatory substances. CPM is the human surrogate measure for diffuse noxious inhibitory control (Yarnitsky, 2010). Yarnitsky et al., 2012 (Yarnitsky et al., 2012) suggested an analgesic effect of duloxetine and a normalization in CPM responses in patients with diabetic neuropathy.

It has been suggested that CPM (Yarnitsky et al., 2012) and heat pain thresholds (Kisler et al., 2019) can predict the analgesic effect of duloxetine and thereby explain the underlying mechanistic mode‐of‐action leading to a possible personalized pain management regime. In addition, cognitive factors are often used to predict treatment responses (Edwards et al., 2011). A recent study found that a combination of preoperative QST, cognitive factors and clinical pain predicted chronic postoperative pain after total knee arthroplasty better than each parameter alone (Larsen, Laursen, Edwards, et al., 2021). This suggests that a multidimensional evaluation of patients prior to OA treatment might be advantageous when pursuing a personalized management approach.

The primary aim of this proof‐of‐mechanism, randomized, placebo‐controlled, double‐blinded, crossover trail was to investigate the effect of an 18‐week duloxetine treatment on mechanistic pain biomarkers in patients with painful OA and the study was therefore not powered to investigate the potential analgesic effect of duloxetine compared to placebo. Secondary aims included (A) the effects of duloxetine on cognitive factors and clinical pain features and (B) an attempt to predict the analgesic response of duloxetine using a combination of pretreatment QST, cognitive factors and clinical pain.

## 2 METHODOLOGY

**FIGURE 1:**
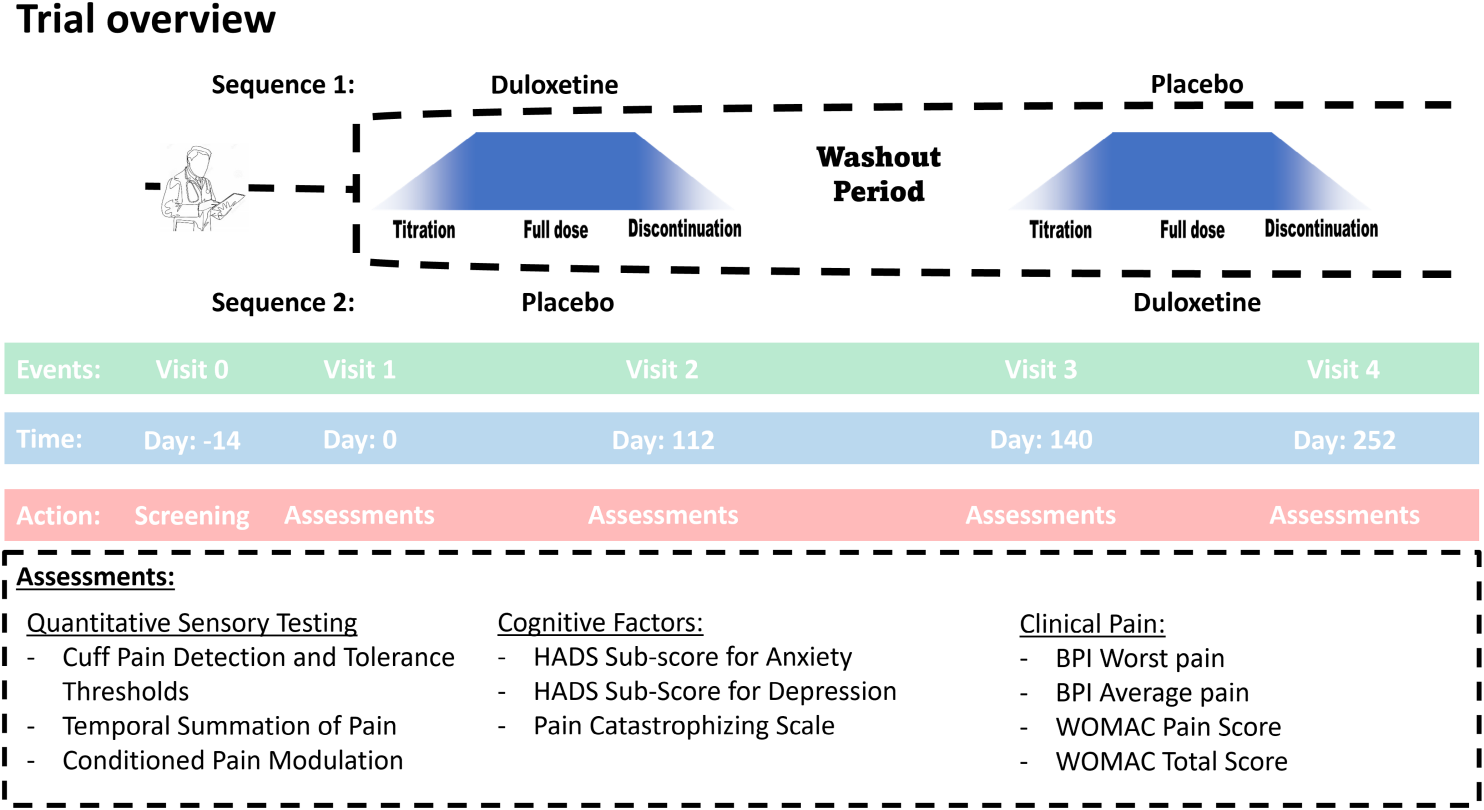
Patients with osteoarthritis were screened (visit 0) and randomized (visit 1) into 18 weeks of duloxetine followed by 18 weeks of placebo (sequence 1) or 18 weeks of placebo followed by 18 weeks of duloxetine (sequence 2). A 2‐week washout period was conducted between the two treatments. Patients were assessed before (visit 1 and 3) and after (visit 2 and 4) each treatment. Assessments were conducted at each of the four visits. HADS: Hospital anxiety and depression scale, BPI: Brief pain inventory, WOMAC: Western Ontario and McMaster universities osteoarthritis index.

This proof‐of‐mechanism, randomized, placebo‐controlled, double‐blinded, crossover trail compared QST (PPTs, TSP and CPM) and cognitive factors (anxiety, depression and pain catastrophizing) before and after 18 weeks of duloxetine and placebo. Forty patients were randomized to one of two equally sized sequences: (1) duloxetine followed by placebo or (2) placebo followed by duloxetine. Patients were screened for inclusion (visit 0) and assessed before (visit 1 and visit 3) and after the treatments (visit 2 and 4); see Figure 1 for overview. Visit 2 and 4 were conducted when the patients received the full dose treatment and before the discontinuation period. Randomization was conducted before baseline measurements at visit 1. Adverse events were assessed at each visit.

The study was approved by The Danish Medicines Agency (case number: 2019082317), The North Denmark Region Committee on Health Research Ethics (case number: N‐20190050), registered at ClinicalTrial.gov (clinicaltrials.gov identifier: NCT04224584), and in the European Union Drug Regulating Authorities Clinical Trials Database (EudraCT number: 2019–003437‐42). The study protocol is published (Ammitzbøll et al., 2021). The trial was continuously monitored by the Good Clinical Practice (GCP) Unit of Aalborg University Hospital, externally audited by the Danish Medicines Agency, and was conducted in accordance with The Helsinki Declaration, GCP, and all applicable Danish regulatory requirements. Informed consent was obtained from all participants.

### 2.1 Study population

Patients were recruited through Synexus/C4Pain, Aalborg, Denmark (a contracting research organization). Women and men, 40–75 years of age, with OA of the knee, who agreed to participate and filled in an informed consent, were included. Patients fulfilling the inclusion criteria were invited to a screening visit (visit 0) during which a physical and psychological (for evaluation of suicidal risk) examination was conducted by a medical doctor. The screening included x‐ray of the knee, screening of medical records and screening of the eligibility criteria. Patients were asked to discontinue all analgesic pain medication (including non‐steroidal anti‐inflammatory drugs [NSAIDs]) during the entire trial, and the patients were instructed to note the use of rescue medication (paracetamol) in a trial‐specific diary.

According to the inclusion and exclusion criteria, patients who were eligible were invited to visit 1 during which they were included in the study and randomized.

### 2.2 Inclusion criteria and exclusion criteria

Patients diagnosed with unilateral or bilateral OA of the knee according to the American College of Rheumatology criteria based on clinical and radiographic evidence (Altman et al., 1986) were recruited. In addition, patients provided written informed consent and abided by the study restrictions. Prior to enrolment, the patients displayed a Kellgren and Lawrence grade of I, II or III at the index knee, reported worst pain intensity within the last 24 h as 5.0 to 10.0 cm (assessed on a 0–10 cm visual analogue scale [VAS] anchored at 0 cm: no pain and 10 cm: worst pain imaginable) and agreed to maintain the same activity level throughout the course of the study.

Enrolment was restricted to people aged 40 years or older because knee pain in younger patients is often due to trauma rather than to naturally occurring OA. Patients were screened for suicidal risk using the Columbia‐Suicide Severity Rating Scale (Posner et al., 2011), and patients at risk were excluded to ensure patient welfare. Interactions with other drugs are potentially a bias. Therefore, patients having specific medical conditions other than OA or taking specific medications other than the allowed were excluded. Patients taking certain psychoactive medications, abusing drugs or alcohol or having other dependencies were excluded because of the potential confounding factors of these medications/substances on the results.

### 2.3 Treatments

Treatment periods included a 2‐week titration period (week 1 (7 days): 20 mg/daily, week 2 (7 days): 40 mg/daily), a 14‐week full treatment period (week 3–16 [70 days]: 60 mg/daily) followed by a 2‐week discontinuation period (week 17 (7 days): 40 mg/daily, week 18 (7 days): 20 mg/daily). The treatment periods were separated by at least 2 weeks (washout period). See Figure 1 for overview.

The patients were instructed to take one capsule of study drug orally with approximately 200 ml of water at room temperature after breakfast in the morning of each dosing day.

This study was initiated before the COVID‐19 pandemic, and all subjects were randomized prior to the lockdown in Denmark. The original protocol aimed for 10 weeks of full treatment of duloxetine and placebo, but this was increased to 14 weeks to allow for study visits during the lockdown period, to minimize suspension of subjects, and to increase the willingness of continued participation.

### 2.4 Blinding and randomization

The study drug was encapsulated in a gelatin capsule (DBCaps® from Capsugel, size: AAEL, colour HPMC Swedish Orange Opaque) with identical size, colour and weight to ensure blinding. The study drug was produced and labelled by the Hospital Pharmacy at Aarhus University Hospital, Aarhus, Denmark. The blinding and randomization procedures were conducted by the Hospital Pharmacy at Aarhus University Hospital. Patients are block‐randomized (four patients at a time) to either sequence 1 (e.g., duloxetine followed by placebo) or sequence 2 (e.g., placebo followed by duloxetine). Patients, study personnel and study management were blinded until the end of the trial. Procedures for unblinding were initiated if the patient safety was at risk.

## 3 ASSESSMENTS

Mechanistic pain profiles, cognitive factors and clinical pain were assessed at visits 1, 2, 3 and 4.

### 3.1 Mechanistic pain profiling

Deep tissue pain sensitivity was evaluated by cuff pressure stimuli using a computer‐controlled cuff algometer (Cortex Technology and Aalborg University), including a 13‐cm wide tourniquet cuff (VBM) and an electronic VAS (Aalborg University) for the recording of the pain intensity. The cuff was placed at the head of the gastrocnemius muscle of the lower leg at the index knee. The electronic continuous VAS (sliding resistor) was 10 cm long and sampled at 10 Hz; 0 cm: no pain and 10 cm: worst pain imaginable. Cuff algometry is a reliable assessment for PPTs, TSP and CPM (Graven‐Nielsen et al., 2017; Imai et al., 2016) and has often been utilized in studies with OA patients (Izumi et al., 2017; Kjær et al., 2019; Petersen et al., 2016; Petersen, Simonsen, et al., 2019).

The pressure of the cuff was increased by 1 kPa/s and the patient was instructed to rate the pain intensity continuously on the electronic VAS until the tolerance level was reached. At this point, the patient was instructed to press a stop button. The pressure pain detection threshold (cPDT) was defined as the pressure at which the VAS score exceeded 1 cm as in previous studies (Kristensen et al., 2021; Larsen, Laursen, Edwards, et al., 2021; Petersen, Simonsen, et al., 2019). The pain tolerance threshold (cPTT) was defined when the patient pressed the stop button. The measurements were conducted once on both the ipsilateral and contralateral lower leg to the most affected knee.

Ten short‐lasting stimuli (1 s each) at the level of the cPTT were given at the lower leg with a 1 s break between stimuli. The participants were instructed to continuously rate the pain intensity of the sequential stimuli using the electronic VAS and not return to zero during the breaks. For each cuff stimulus, a VAS score was extracted. TSP was calculated as the absolute difference between the last three stimuli and the first three stimuli as in previous studies (Petersen, Olesen, et al., 2019; Staffe et al., 2019).

The CPM magnitude was assessed as the absolute changes in cPDT with and without a cuff conditioning stimulus. The conditioning stimulus was applied to the contralateral lower leg, and the cPDT was assessed on the ipsilateral lower leg as described above. The conditioning stimulus was applied as a constant stimulus with an intensity of 70% of the pain tolerance level on the contralateral leg (Graven‐Nielsen et al., 2017; Petersen, Andersen, et al., 2018; Petersen, Simonsen, et al., 2019). The CPM effect was calculated as the absolute difference in conditioned and unconditioned cPDT (i.e., cPDTconditioned minus cPDTunconditioned).

### 3.2 Cognitive factors

Anxiety and depression symptoms were assessed using the Hospital Anxiety and Depression Scale (HADS) (36), which applies a subscale for anxiety and a subscale for depression. The HADS ranges from 0 to 21; 0 to 7 indicate no symptoms of anxiety/depression, 8 to 10 indicate probable symptoms of anxiety/depression and 11 to 21 indicate potential symptoms of anxiety/depression (Zigmond & Snaith, 1983).

The Pain Catastrophizing Scale (PCS) consists of 13 items focusing on thoughts and feelings in connection with pain (Sullivan et al., 1995). The questions are rated on a 4‐point scale ranging from 0 (not at all) to 3 (very much).

### 3.3 Clinical pain intensity

Clinical pain intensity was assessed using the Brief Pain Inventory (BPI). The worst pain and average pain within the last 24 h were evaluated using a 10 cm VAS anchored at 0 cm: no pain, and 10 cm: worst pain imaginable.

Additionally, clinical pain was assessed using the Western Ontario and McMaster Osteoarthritis Scale (WOMAC) (Bellamy et al., 1988), a patient‐rated instrument that measures OA symptoms. The questionnaire contains five pain questions, two stiffness questions and 17 physical function questions (24 questions in total). Each question utilizes a 5‐point scale from 0 (none) to 4 (extreme). Both the WOMAC pain scale and the total WOMAC score were used in the analysis.

### 3.4 Sample size

Wang et al., 2019 [2] demonstrated an effect size of 0.55 when assessing the worst pain within the last 24 h for a 10‐week treatment of duloxetine compared with placebo in a parallel design with patients with moderate‐to‐severe OA. We hypothesized that the analgesic effect of duloxetine acted through modulation of serotonin and noradrenaline, which would act on the descending pain inhibitory pathways and thereby provide modulation in QST. Therefore, a sample equation with 85% power and a significant level at 0.05 using a crossover design yielded 32 patients, which we assumed would be sufficient to detect a modulation in the QST parameters. Forty patients were enrolled to account for potential dropouts.

### 3.5 Statistical analysis

Repeated measures analysis of variances (RM‐ANOVAs) with factors time (before and after treatments) and drug (placebo or duloxetine) were conducted for all mechanistic pain profiling methods, cognitive factors and clinical pain parameters to investigate significant differences. Additionally, paired sample *t*‐tests were used to evaluate absolute and percentage changes in clinical pain for each treatment. The Bonferroni post hoc test was utilized to adjust for multiple comparisons. Responders to treatment were classified based on a 30% and 50% pain reduction in clinical pain comparing before and after treatments and compared using the Fisher's exact Test.

Several linear regression models were used to predict the analgesic effect (dependent variables: mean change in BPI worst pain, BPI average pain, WOMAC pain and WOMAC total score) of duloxetine and placebo using the mechanistic pain profiles, cognitive factors and clinical pain prior to treatment. Backward elimination was applied to the linear regressions to identify independent predictors using cut‐offs for statistical independence and inclusion of 0.05 and exclusion of 0.157, respectively, according to Akaike's information criterion for prognostic models (Heinze & Dunkler, 2017). The standardized beta coefficient (normalized beta coefficients to a standard deviation for easier comparisons between different variable) will be reported for each independent parameter. A higher standardized beta coefficient indicates a stronger association to the dependent variable.

Statistical tests were conducted using the IBM SPSS Statistics software version 26 (IBM). A value of *p* < 0.05 was considered a significant finding. All data are presented as means ± standard deviation (SD) unless otherwise specified.

### 3.6 Availability of data and materials

The data used and analysed are available by contacting the corresponding author on reasonable request.

## 4 RESULTS

### 4.1 Patient flow

The first patient was assessed at the first visit on 18 December 2019, and the last patient was assessed at the last visit on 1 July 2021. Administration of the study drug was postponed for two patients in sequence 1, and two patients in sequence 2 due to the COVID‐19 lockdown of Denmark. Later, these four patients withdrew their consent and hence were never administrated any study drug.

In sequence 1, three patients discontinued the study in treatment period 1 (duloxetine treatment) due to side effects: one patient experienced psychological issues, one patient experienced constipation, and one patient experienced impaired ejaculation.

In sequence 2, two patients discontinued the study in treatment period 1 (placebo treatment) due to side effects: one patient experienced signs of liver cirrose (with dark urine), and one patient experienced dizziness. Additionally, one patient experienced a loss of libido in treatment period 2 (duloxetine treatment).

**FIGURE 2:**
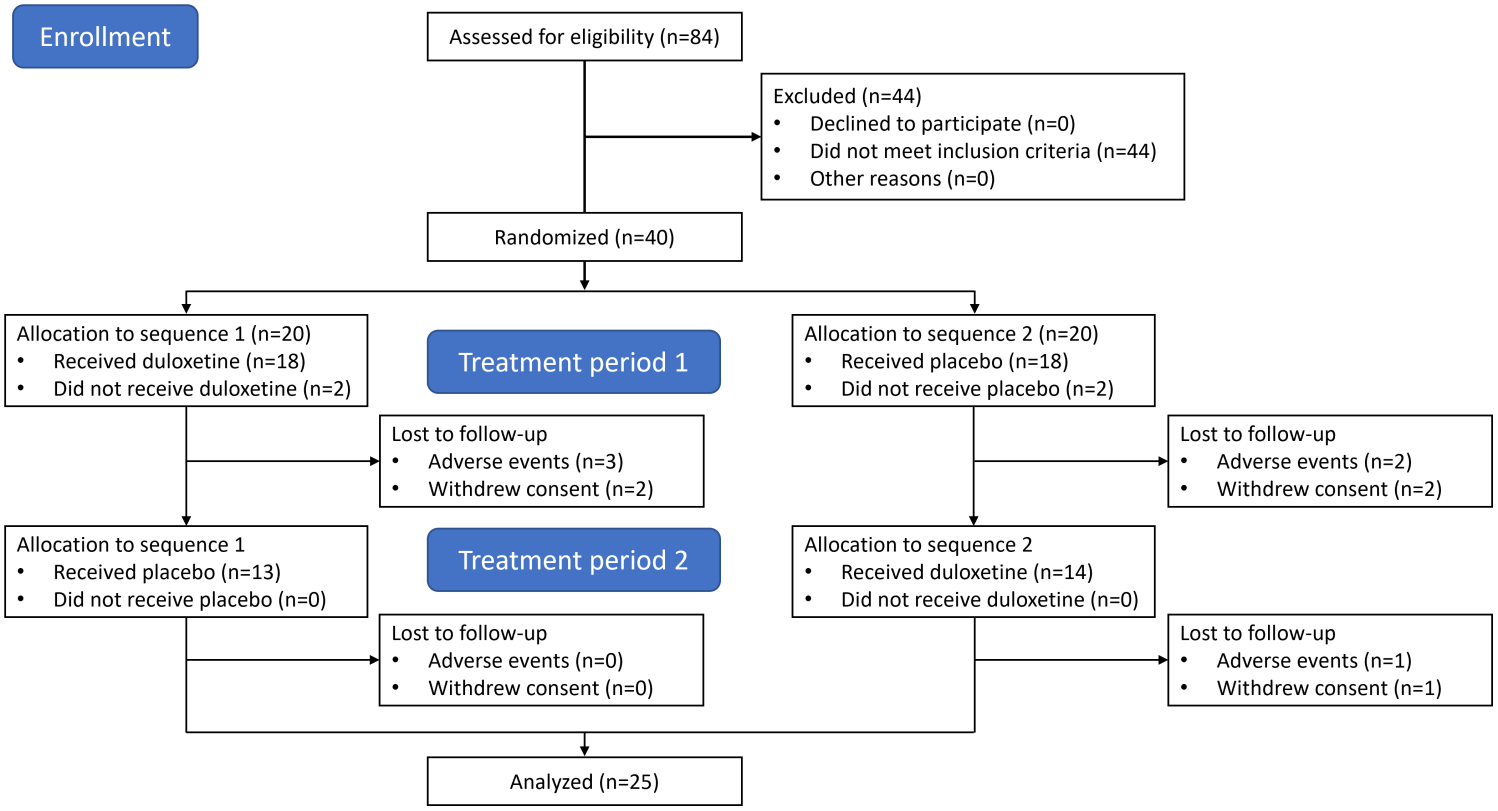
CONSORT diagram of patient flow.

Twenty‐five patients completed all the visits and had full data for the analysis, see Table 1 for demographic information on the patients who completed the trial and Figure 2 for CONSORT diagram.

**TABLE 1:** Baseline data from patients completing the trial

Age (mean ± SD) (years)	65.12 (7.18)
BMI (mean ± SD) (kg/m^2^)	27.94 (3.73)
Pain severity (mean ± SD) (VAS)	6.64 (1.55)
Gender (females/males)	18/7
Kellgren and Lawrence grading (mean ± SD)	2.10 (0.77)

Abbreviations: BMI, Body Mass Index; SD, Standard deviation; VAS, Visual analogue scale.

### 4.2 Non‐responder analysis

No significant differences were found comparing patients who completed the trial and patients who did not complete the trial regarding baseline pain severity (independent *t*‐test: *p* = 0.96), age (independent *t*‐test: *p* = 0.83), body mass index (independent *t*‐test: *p* = 0.60), Kellgren and Lawrence grading (independent *t*‐test: *p* = 0.77) or gender distribution (Chi‐square: *p* = 0.15).

### 4.3 Adverse events

Significantly more adverse events occurred during the duloxetine treatment (average: 2.28, SD: 1.54) compared with placebo treatment (1.40, SD: 1.32, paired‐sample t‐test: *p* = 0.03). The most common adverse events during the duloxetine treatment were gastroenterological (58% of patients), neurological (44% of patients), dry mouth and nausea (40% of patients) and fatigue (28% of patients). The most common adverse events during the placebo treatment were neurological (36% of patients), gastroenterological (24% of patients), dry mouth and nausea (24% of patients) and fatigue (16% of patients).

One serious adverse event occurred during the duloxetine treatment period where one patient experienced severe headache, palpitations and difficulty breathing, and the patient was hospitalized for 1 day to ensure safety. Later it was discovered that this patient had a medical history of cardiovascular problems and had received bypass surgery 4–5 years prior to the current study. This patient did not withdraw the consent and continued in the study.

### 4.4 Modulation of pain mechanisms

**FIGURE 3:**
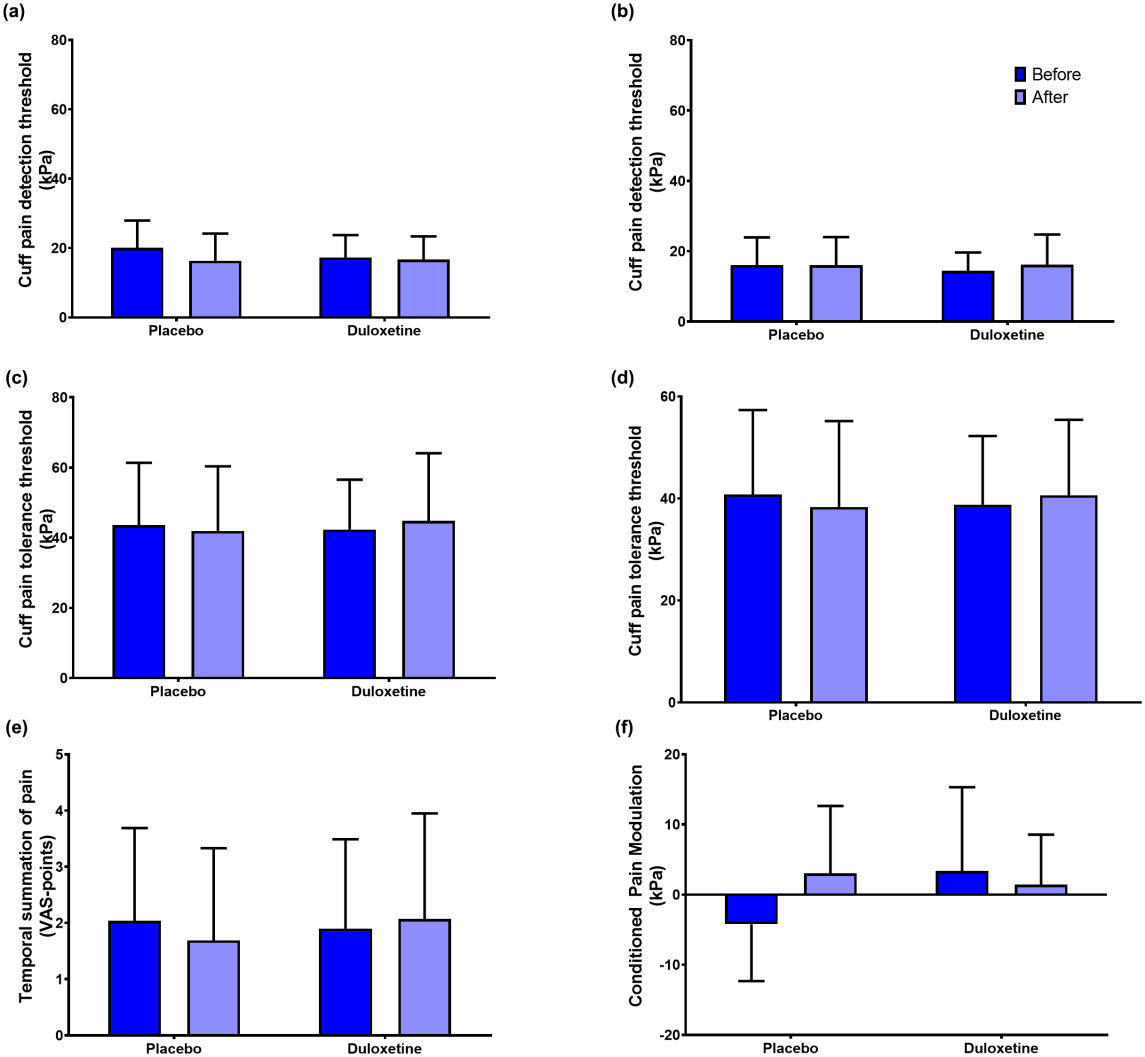
Quantitative sensory testing data before (dark blue) and after (light blue) 18 weeks' treatment with either placebo or duloxetine. QST was assessed using ipsilateral (a) and contralateral (b) cuff pain detection thresholds, ipsilateral (c) and contralateral (d) cuff pain tolerance thresholds, (e) temporal summation of pain and (f) conditioned pain modulation. The data presented illustrate means and standard deviations.

Significant changes over time were observed for cPDT assessed at the ipsilateral side (F[1,24] = 13.112, *p* = 0.001) and CPM (F[1,24] = 7.407, *p* = 0.013), but no drug effect was seen for cPDT (F[1,24] = 1.110, *p* = 0.303) and CPM (F[1,24] = 1.954, *p* = 0.177). No time or drug effect was seen for cPDT at the contralateral side, cPTT at the ipsilateral and contralateral side, and TSP (F[1,24] < 0.080, *p* > 0.190). See Figure 3.

### 4.5 Modulation of cognitive factors

**FIGURE 4:**
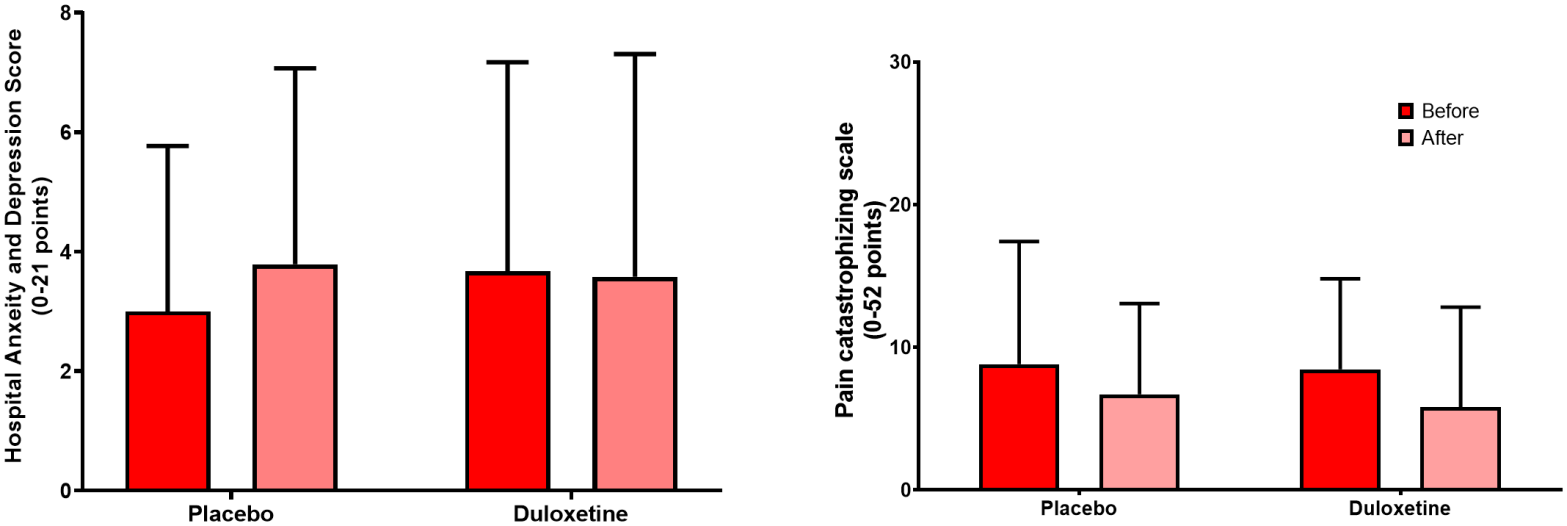
Cognitive factors assessed before (red) and after (pink) 18 weeks' treatment with either placebo or duloxetine. Cognitive factors assessed using (a) the hospital anxiety and depression score and (b) the pain catastrophizing scale. The data presented illustrate means and standard deviations.

No significant different time or treatment effects were found for HADS (F[1,24] < 0.93, *p* > 0.35) and PCS (F[1,24] < 3.92, *p* > 0.060). A trend towards a significant time effect was found for PCS (*p* = 0.06), but the comparing treatments were not different (*p* = 0.39), see Figure 4.

### 4.6 Analgesic effect on clinical pain

No drug effects were seen for the BPI subscale for worst pain (F[1,24] > 0.001, *p* = 1.00), the BPI subscale for average pain (F[1,24] = 0.600, *p* = 0.45), WOMAC pain (F[1,24] = 0.019, *p* = 0.89) and WOMAC total (F[1,24] > 0.001, *p* = 0.99); neither for absolute nor percentage differences. See Table 2.

**TABLE 2:** Differences in treatment responses to duloxetine and placebo treatments and calculated analgesic effects presented as absolute and percentage differences

	Duloxetine		Placebo		*p*‐value	
	Before	After	Before	After	Time	Drug
Time effects of treatments						
BPI worst pain	5.56 (2.29)	3.08 (2.71)	4.92 (2.55)	3.96 (2.48)	*p* < 0.001	1.000
BPI average pain	4.42 (1.95)	2.42 (2.02)	4.00 (2.23)	3.25 (2.09)	*p* < 0.001	0.447
WOMAC pain	8.56 (2.97)	6.44 (3.79)	8.29 (3.58)	6.83 (3.82)	*p* = 0.002	0.890
WOMAC total	36.56 (13.91)	28.28 (19.58)	36.58 (18.01)	30.00 (16.68)	*p* < 0.001	0.994

			Mean difference	*p*‐value
Absolute differences comparing treatments				
BPI Worst pain	2.48 (3.38)	1.28 (2.49)	1.20 (4.74)	0.218
BPI average pain	1.96 (2.85)	0.96 (2.41)	1.00 (4.20)	0.246
WOMAC pain	2.12 (4.14)	1.84 (4.12)	0.28 (6.255)	0.825
WOMAC total	8.28 (18.96)	8.48 (14.91)	0.20 (27.93)	0.972

			Mean difference	*p*‐value
Percentage differences comparing treatments				
BPI Worst pain	33.69 (59.51)	16.98 (52.31)	15.37 (78.86)	0.340
BPI Average pain	36.84 (65.75)	2.08 (111.95)	34.77 (123.51)	0.266
WOMAC pain	21.02 (44.31)	21.30 (46.95)	0.28 (58.96)	0.981
WOMAC total	20.02 (48.21)	23.29 (35.34)	3.27 (58.66)	0.783

Abbreviations: BPI, Brief Pain Inventory; WOMAC, Western Ontario and McMaster Universities Osteoarthritis Index.

### 4.7 Responders

Number of patients who had a reduction in clinical pain by 30% or 50% are presented in Table 3.

**TABLE 3:** Number of patients achieving at least a reduction of 30% or 50% in clinical pain after duloxetine and placebo treatment. The numbers in the parentheses represent the percentage of the full cohort

	Duloxetine	Placebo	*p*‐value
Based on 30% responder criteria			
BPI Worst pain	16 (64%)	11 (44%)	1.000
BPI Average pain	17 (68%)	7 (28%)	0.640
WOMAC pain	10 (40%)	11 (44%)	0.697
WOMAC total	11 (44%)	7 (28%)	1.000
Based on 50% responder criteria			
BPI Worst pain	11 (44%)	6 (24%)	1.000
BPI Average pain	12 (48%)	5 (20%)	0.645
WOMAC pain	6 (24%)	5 (20%)	0.070
WOMAC total	8 (32%)	4 (16%)	1.000

Abbreviations: BPI, Brief Pain Inventory; WOMAC, Western Ontario and McMaster Universities Osteoarthritis Index.

### 4.8 Predicting analgesic response to duloxetine and placebo

Multiple linear regression was constructed to predict the analgesic response to the duloxetine and placebo treatment. Model 1 contained all QST methods, cognitive factors and clinical pain data, whereas Model 2 was developed using the backward elimination.

Depending on the outcome measure, the predictive values ranged from 21.3% (WOMAC pain) to 70.3% (BPI average pain) for Model 1 and 45.7% (WOMAC total) to 75.4% (BPI worst pain) for Model 2 for the duloxetine treatment, see Table 4. These models indicate that reducing the number of predictors increased the predictive value (i.e., from Model 1 to Model 2) and find that a combination of QST measures, cognitive factors and clinical pain data consistently predicted the analgesic effect of duloxetine.

**TABLE 4:** Multiple linear regression models aiming to establish the adjusted predictive value (R^2^) for baseline parameters predicting analgesic response to duloxetine treatment. Model 1 contains all baseline parameters, whereas Model 2 was constructed using backwards selection and aimed to identify independent predictors (bold numbers in Model 2 are significant independent predictors)

Model 1	Standardized β‐values			
	BPI worst pain	BPI average pain	WOMAC pain	WOMAC total
Adjusted R^2^	61.8%	70.3%	21.3%	25.3%
cPDT (ipsi)	0.021	−0.168	−0.084	−0.274
cPDT (contra)	−0.103	−0.404	−0.529	−0.701
cPTT (ipsi)	−0.013	0.411	0.114	0.718
cPTT (contra)	−0.192	−0.223	−0.043	−0.303
TSP	−0.215	−0.033	−0.102	−0.069
CPM	0.080	−0.026	0.153	−0.039
BPI Worst pain	0.996	0.158	0.134	−0.001
BPI Average pain	−0.047	0.665	−0.030	0.080
WOMAC pain	0.091	0.301	0.879	0.250
WOMAC total	−0.315	−0.286	−0.235	0.644
HADS	−0.610	−0.441	−0.530	−0.675
PCS	−0.038	0.006	0.072	0.045

Model 2	Standardized β‐values			
	Worst pain	Average pain	WOMAC pain	WOMAC total
Adjusted R^2^	75.6%	75.3%	48.6%	45.7%
cPDT (ipsi)				
cPDT (contra)		**−0.342**	**−0.491**	**−0.538**
cPTT (ipsi)				
cPTT (contra)	−0.237			
TSP	−0.240*			
CPM				
BPI Worst pain	**0.999**			
BPI Average pain		**0.743**		
WOMAC pain			**0.787**	
WOMAC total	−0.255			**0.781**
HADS	**−0.611**	**−0.441**	**−0.515**	**−0.650**
PCS				

Abbreviations: BPI, Brief Pain Inventory; contra, contralateral side to the most osteoarthritic affected knee; cPDT, cuff pain detection threshold; CPM, conditioned pain modulation; cPTT, cuff pressure pain tolerance threshold; HADS, Hospital Anxiety and Depression Score; ipsi, ipsilateral side to the most osteoarthritic affected knee; PCS, Pain Catastrophizing Scale; TSP, temporal summation of pain; WOMAC, Western Ontario and McMaster Universities Osteoarthritis Index.

Additionally, depending on the outcome measure, the predictive values ranged from 4.8% (WOMAC total) to 20.5% (WOMAC pain) for Model 1 and 22.0% (BPI worst pain) to 41.8% (WOMAC pain) for Model 2 for the placebo treatment, see supplementary Table 1. These models consistently demonstrated that significant independent predictors of the analgesic placebo response were pretreatment clinical pain data.

## 5 DISCUSSION

Depending on the clinical pain outcome parameter, 40%–68% of patients received at least 30% clinical pain reduction and 24%–48% of patients received at least 50% clinical pain reduction during the duloxetine treatment. Depending on the clinical pain outcome parameter, pretreatment mechanistic pain profiles, cognitive factors and clinical pain predicted the analgesic response of duloxetine by 45% to 75%, indicating that patients with high pain sensitivity, higher scores on pain catastrophizing, anxiety and/or depression and higher clinical pain may potentially benefit more from duloxetine treatment. The trial demonstrated no significant changes in stand‐alone mechanistic pain biomarkers, cognitive factors or clinical pain comparing 18 weeks of duloxetine with placebo in patients with painful knee osteoarthritis.

### 5.1 Predicting the analgesic effect of duloxetine

Accumulating evidence suggests that QST parameters can predict OA treatment responses to, for example, total joint replacement surgeries (Izumi et al., 2017; Kurien et al., 2018; Larsen, Laursen, Edwards, et al., 2021; Petersen et al., 2015, 2016; Petersen, Simonsen, et al., 2018), NSAIDs (Arendt‐Nielsen et al., 2016; Edwards et al., 2016; Petersen, Olesen, et al., 2019; Petersen, Simonsen, et al., 2019), or exercise interventions (Hansen et al., 2020; O'Leary et al., 2018). Similarly, it seems evident that cognitive factors are associated with treatment outcomes in patients with OA (Brander et al., 2007; Edwards et al., 2011; Escobar et al., 2007; Forsythe et al., 2008; Kendell et al., 2001). A recent study demonstrated that a combination of preoperative mechanistic pain biomarkers and assessment of cognitive factors predicted chronic postoperative pain after total knee replacement surgery better than each of the factors alone (Larsen, Laursen, Edwards, et al., 2021). Similarly, the current study demonstrated that a combination of mechanistic pain biomarkers, cognitive factors, and clinical pain consistently predicted the analgesic response of duloxetine, and this is independent of the outcome parameters. Composite outcomes merge two or more relevant clinical outcomes into a single measure and Gewandter et al., (Gewandter et al., 2021) recently highlighted that composite outcomes, rather for example, clinical pain intensity, should be utilized in clinical pain trials, since the composite outcomes are more clinical relevant when addressing multifactorial disorders such as chronic pain. The current trial supports that a multidimensional assessment of relevant pain parameters prior to treatment is needed to ensure a stronger prediction of treatment outcomes. Hence, this may be an avenue to pursue in the attempt to develop personalized pain management regimes.

### 5.2 Modulation of mechanistic pain profiles

It has been argued that pain intensity and pain duration are associated with lowering of pressure pain thresholds, facilitation of TSP and impairment of CPM (Arendt‐Nielsen et al., 2010; Arendt‐Nielsen & Graven‐Nielsen, 2011; Petersen, 2021), which are often seen in patients with severe OA when compared with healthy pain‐free subjects (Arendt‐Nielsen et al., 2015). Based on this, studies have argued that pain relief from, for example, total joint replacement or NSAIDs is associated with normalization of QST parameters in patients with OA (Arendt‐Nielsen et al., 2016; Graven‐Nielsen et al., 2012; Kosek & Ordeberg, 2000), but conflicting evidence does exist (Petersen et al., 2015; Petersen, Simonsen, et al., 2019). The current study did not find significant analgesic effects (by assessing single parameters) comparing duloxetine and placebo treatments, and this could be associated with the lack of modulation of the assessed pain mechanisms. However, it also argues for the importance of combining different outcome parameters as end‐points in clinical trials.

Pre‐clinical evidence suggests that serotonin and noradrenalin are important neurotransmitters for the descending pain inhibitory pathways (Bannister et al., 2015; Bannister & Dickenson, 2017; Lockwood et al., 2019). This has been used to explain the mode‐of‐action of duloxetine since duloxetine modulates these neurotransmitters. Yarnitsky et al., 2012 (Yarnitsky et al., 2012) demonstrated, in a non‐placebo controlled study, an analgesic effect of duloxetine in patients with diabetic neuropathy and found duloxetine to improve CPM. Similarly, Kisler et al., 2019 (Kisler et al., 2019) demonstrated that duloxetine improved CPM in patients with migraine. The current study is the first study to evaluate the effect of duloxetine on QST parameters in patients with knee OA. As our data are, for the single outcome parameters, did not support the previous suggested mechanistic actions the explanation could be that the QST methodologies utilized in the previous studies (Kisler et al., 2019; Yarnitsky et al., 2012) are different from those in the current study. Furthermore our study is a GCP‐monitored, randomized, placebo‐controlled, crossover, double‐blinded trial utilizing QST methodology with good‐to‐excellent reliability (Graven‐Nielsen et al., 2017; Imai et al., 2016). The data from the current study are also similar to other OA studies (Kurien et al., 2018; Larsen, Laursen, Edwards, et al., 2021; Petersen, Olesen, et al., 2019; Petersen, Simonsen, et al., 2019) with the same QST methodology used. Studies using this QST methodology have demonstrated that the QST parameters are modifiable by, for example, exercise‐based therapy in both OA (Holm et al., 2021) and non‐OA patient cohorts (Heredia‐Rizo et al., 2019). Additionally, the previous studies utilized treatment periods of 4 weeks (Yarnitsky et al., 2012) and 8 weeks (Kisler et al., 2019), which are shorter than the current study. It is, however, important to acknowledge that inter‐individual variability is a valid concern for QST assessments (Kennedy et al., 2016) and it is difficult to conclude if the variability of modulation in mechanistic pain profiles is due to methodological differences, due to the relatively low number of trials published within this area, or due to the relative small sample sizes.

### 5.3 Modulation of cognitive factors

Duloxetine is a treatment for major depression, and studies have demonstrated that a subsample of patients with severe knee OA demonstrates signs of depression and anxiety (Larsen, Laursen, Simonsen, et al., 2021). Therefore, it would be logical to assume that the analgesic effect of duloxetine was due to improvement in signs of depression and anxiety. Bjelland et al., 2002 (Bjelland et al., 2002) reviewed the studies on the validity of the HADS questionnaire to identify anxiety and depression and found a cut‐off point of 8/21 on each subscale to identify anxiety and depression. The current study demonstrated average HADS anxiety and depression scores below 8/21. Therefore, this cohort might not have been ideal for the antidepressant properties of duloxetine. In contrast to this, Chappell et al., 2009 (Chappell et al., 2009) argued that the analgesic effect of duloxetine for OA pain is not dependent on improvements of anxiety and depression scores but that duloxetine acts a purely analgesic substance. However, this was not supported by the current trial.

### 5.4 The analgesic effect of duloxetine in osteoarthritis

Seven studies have consistently demonstrated significant clinical analgesic effects of duloxetine for OA pain (Abou‐Raya et al., 2012; Chappell et al., 2009, 2011; De Tommaso et al., 2007; Frakes et al., 2011; Uchio et al., 2018; Wang et al., 2019) with a daily dose varying from 60 mg/daily (Abou‐Raya et al., 2012; Uchio et al., 2018; Wang et al., 2015) up to 120 mg/daily (Chappell et al., 2009, 2011; Frakes et al., 2011). Similar to the current study, the previous studies included patients with KL grade I‐III, moderate‐to‐severe pain intensity levels, and OA duration for a minimum of 3 years (Chen et al., 2021). The treatment outcomes of the different trials varied, but four trials utilized a combination of the BPI and WOMAC to assess the analgesic effect (Chappell et al., 2009, 2011; Frakes et al., 2011; Wang et al., 2019), which is similar to the current trial.

Recent studies have included an enrichment strategy for recruiting patients based on higher scores on the PainDetect Questionnaire (De Tommaso et al., 2007) or the Central Sensitization Inventory (Koh et al., 2019), which have demonstrated analgesic effects when compared to placebo. However, a recent RCT utilized PainDetect Questionnaire for an enriched patient population but did not demonstrate significant analgesic effects of duloxetine when compared to placebo (Rienstra et al., 2021). The current study did not implement an enrichment strategy, which could, in part, explain the lack of analgesic effect in the current trial.

The current study was not powered towards demonstrating an analgesic effect but rather to demonstrate an effect on the mechanistic pain profiles. Previous studies have demonstrated approx. 1 point mean difference in analgesic effect scores when comparing duloxetine and placebo using the BPI pain score (Chappell et al., 2009, 2011; Frakes et al., 2011; Uchio et al., 2018; Wang et al., 2015), which is similar to the current trial.

### 5.5 Limitation

All patients were enrolled before the COVID‐19 lock‐down in Denmark (March 13, 2020), but the treatment periods were conducted during the COVID‐19 pandemic and lock‐down. Studies have demonstrated that COVID‐19 negatively impacted anxiety, depression, and psychological stress in the general population and in different patient groups (Jin et al., 2021; Momenimovahed et al., 2021), which could have impacted the results.

A total of 32 patients should complete this trial to reach a power of 85%. Forty patients were enrolled to ensure a sufficient margin of error in case of dropouts, but only 25 patients completed the study. Therefore, the current analysis is underpowered, and the results should be cautiously interpreted. Additionally, the current trial was not powered to investigate a potential analgesic effect of duloxetine when compared to placebo and this should be considered when interpreting the results.

The current study does indicate that mechanistic pain profiling, cognitive factors and clinical pain can predict the analgesic response of duloxetine and therefore it would be interesting to conduct exploratory analysis of subgroup of patients responding and not responding to the duloxetine treatment. This is analysis is not possible in the current study due to the low sample size but should be conducted in future studies.

## 6 CONCLUSION

This proof‐of‐mechanism, randomized, placebo‐controlled, double‐blinded, crossover GCP‐monitored trial found that 40%–68% of patients received at least 30% pain reduction, and 24%–48% of patients received at least 50% clinical pain reduction by the duloxetine treatment, which was depending on the clinical pain outcome parameter to assess the analgesic effect. Depending on the clinical pain outcome parameter, pretreatment mechanistic pain profiling, cognitive factors and clinical pain predicted the analgesic response of duloxetine with a prediction value of 47%–75%. No group‐level significant changes in single, stand‐alone clinical pain parameters, mechanistic pain biomarkers, or cognitive factors were found when 18 weeks of duloxetine was compared to placebo in patients with painful knee osteoarthritis. This trial demonstrates that only a subsample of patients with severe OA gained an analgesic benefit of duloxetine and that these patients could be stratified based on mechanistic pain profiling, cognitive factors and clinical pain parameters and hence may give indications for developing personalized pain management regimes.

### AUTHOR CONTRIBUTION

KKP, AMD, AEO and LAN conceptualized the design of the study, wrote the protocol and initiated the trial. KKP, AMD, DB, CB and NA implemented the protocol, communicated with the Regulatory Authorities and conducted the study. KKP analysed the data and wrote the first draft of the manuscript, and all authors critically revised the manuscript and approved the final version.

### FUNDING INFORMATION

Center for Neuroplasticity and Pain (CNAP) is supported by the Danish National Research Foundation (DNRF121). The Aalborg University Talent Management Programme (j.no. 771126), The Danish Rheumatism Association (grant number: A6579), The Shionogi Science Program, and the TaNeDS Daiichi Sankyo Science Center Europe grant funded the study.

### CONFLICT OF INTEREST

The authors declare no conflict of interest.

## Supporting information


Table S1

## References

[ejp1988-bib-0001] Abou‐Raya, S. , Abou‐Raya, A. , & Helmii, M. (2012). Duloxetine for the management of pain in older adults with knee osteoarthritis: Randomised placebo‐controlled trial. Age and Ageing, 41, 646–652. 22743149 10.1093/ageing/afs072

[ejp1988-bib-0002] Altman, R. , Asch, E. , Bloch, D. , Bole, G. , Borenstein, D. , Brandt, K. , Christy, W. , Cooke, T. D. , Greenwald, R. , Hochberg, M. , Howell, D. , Kaplan, D. , Koopman, W. , Longley, S. , Mankin, H. , McShane, D. J. , Medsger, T. , Meenan, R. , Mikkelsen, W. , … Wolfe, F. (1986). Development of criteria for the classification and reporting of osteoarthritis: Classification of osteoarthritis of the knee. Arthritis and Rheumatism, 29, 1039–1049. 3741515 10.1002/art.1780290816

[ejp1988-bib-0003] Ammitzbøll, N. , Arendt‐Nielsen, L. , Bertoli, D. , Brock, C. , Olesen, A. E. , Kappel, A. , Drewes, A. M. , & Petersen, K. K. (2021). A mechanism‐based proof of concept study on the effects of duloxetine in patients with painful knee osteoarthritis. Trials, 22, 1–11. 34961547 10.1186/s13063-021-05941-y PMC8710922

[ejp1988-bib-0004] Arendt‐Nielsen, L. , Egsgaard, L. L. , & Petersen, K. K. (2016). Evidence for a central mode of action for etoricoxib (COX‐2 inhibitor) in patients with painful knee osteoarthritis. Pain, 157, 1634–1644. 27007068 10.1097/j.pain.0000000000000562

[ejp1988-bib-0005] Arendt‐Nielsen, L. , & Graven‐Nielsen, T. (2011). Translational musculoskeletal pain research. Best Practice & Research: Clinical Rheumatology, 25, 209–226. 22094197 10.1016/j.berh.2010.01.013

[ejp1988-bib-0006] Arendt‐Nielsen, L. , Nie, H. , Laursen, M. B. , Laursen, B. S. , Madeleine, P. , Simonsen, O. H. , & Graven‐Nielsen, T. (2010). Sensitization in patients with painful knee osteoarthritis. Pain, 149, 573–581. 20418016 10.1016/j.pain.2010.04.003

[ejp1988-bib-0007] Arendt‐Nielsen, L. , Skou, S. T. , Nielsen, T. A. , & Petersen, K. K. (2015). Altered central sensitization and pain modulation in the CNS in chronic joint pain. Current Osteoporosis Reports, 13, 225–234. 26026770 10.1007/s11914-015-0276-x

[ejp1988-bib-0008] Bannister, K. , & Dickenson, A. H. (2017). The plasticity of descending controls in pain: Translational probing. The Journal of Physiology, 595, 4159–4166. 28387936 10.1113/JP274165 PMC5491855

[ejp1988-bib-0009] Bannister, K. , Lockwood, S. , Goncalves, L. , Patel, R. , & Dickenson, A. H. (2017). An investigation into the inhibitory function of serotonin in diffuse noxious inhibitory controls in the neuropathic rat. European Journal of Pain, 21, 750–760. 27891703 10.1002/ejp.979

[ejp1988-bib-0010] Bannister, K. , Patel, R. , Goncalves, L. , Townson, L. , & Dickenson, A. H. (2015). Diffuse noxious inhibitory controls and nerve injury: Restoring an imbalance between descending monoamine inhibitions and facilitations. Pain, 156, 1803–1811. 26010460 10.1097/j.pain.0000000000000240

[ejp1988-bib-0011] Bannuru, R. R. , Osani, M. C. , Vaysbrot, E. E. , Arden, N. K. , Bennell, K. , Bierma‐Zeinstra, S. M. A. , Kraus, V. B. , Lohmander, L. S. , Abbott, J. H. , Bhandari, M. , Blanco, F. J. , Espinosa, R. , Haugen, I. K. , Lin, J. , Mandl, L. A. , Moilanen, E. , Nakamura, N. , Snyder‐Mackler, L. , Trojian, T. , … McAlindon, T. E. (2019). OARSI guidelines for the non‐surgical management of knee, hip, and polyarticular osteoarthritis. Osteoarthritis and Cartilage, 27, 1578–1589. 31278997 10.1016/j.joca.2019.06.011

[ejp1988-bib-0012] Bellamy, N. , Buchanan, W. W. , Goldsmith, C. H. , Campbell, J. , & Stitt, L. W. (1988). Validation study of WOMAC: A health status instrument for measuring clinically important patient relevant outcomes to antirheumatic drug therapy in patients with osteoarthritis of the hip or knee. The Journal of Rheumatology, 15, 1833–1840. 3068365

[ejp1988-bib-0013] Berenbaum, F. , Wallace, I. J. , Lieberman, D. E. , & Felson, D. T. (2018). Modern‐day environmental factors in the pathogenesis of osteoarthritis. Nature Reviews Rheumatology, 14, 674–681. 30209413 10.1038/s41584-018-0073-x

[ejp1988-bib-0014] Bjelland, I. , Dahl, A. A. , Haug, T. T. , & Neckelmann, D. (2002). The validity of the hospital anxiety and depression scale. Journal of Psychosomatic Research, 52, 69–77. 11832252 10.1016/s0022-3999(01)00296-3

[ejp1988-bib-0015] Blikman, T. , Rienstra, W. , van Raaij, T. M. , ten Hagen, A. J. , Dijkstra, B. , Zijlstra, W. P. , Bulstra, S. K. , Stevens, M. , & van den Akker‐Scheek, I. (2022). Duloxetine in OsteoArthritis (DOA) study: Effects of duloxetine on pain and function in end‐stage hip and knee OA – A pragmatic enriched randomized controlled trial. BMC Musculoskeletal Disorders, 23, 115. 35123461 10.1186/s12891-022-05034-0 PMC8818142

[ejp1988-bib-0016] Brander, V. , Gondek, S. , Martin, E. , & Stulberg, S. D. (2007). Pain and depression influence outcome 5 years after knee replacement surgery. Clinical Orthopaedics and Related Research, 464, 21–26. 17603386 10.1097/BLO.0b013e318126c032

[ejp1988-bib-0017] Chappell, A. S. , Desaiah, D. , Liu‐Seifert, H. , Zhang, S. , Skljarevski, V. , Belenkov, Y. , & Brown, J. P. (2011). A double‐blind, randomized, placebo‐controlled study of the efficacy and safety of duloxetine for the treatment of chronic pain due to osteoarthritis of the knee. Pain Practice, 11, 33–41. 20602715 10.1111/j.1533-2500.2010.00401.x

[ejp1988-bib-0018] Chappell, A. S. , Ossanna, M. J. , Liu‐Seifert, H. , Iyengar, S. , Skljarevski, V. , Li, L. C. , Bennett, R. M. , & Collins, H. (2009). Duloxetine, a centrally acting analgesic, in the treatment of patients with osteoarthritis knee pain: A 13‐week, randomized, placebo‐controlled trial. Pain, 146, 253–260. 19625125 10.1016/j.pain.2009.06.024

[ejp1988-bib-0019] Chen, B. , Duan, J. , Wen, S. , Pang, J. , Zhang, M. , Zhan, H. , & Zheng, Y. (2021). An updated systematic review and meta‐analysis of duloxetine for knee osteoarthritis pain. The Clinical Journal of Pain, 37, 852–862. 34483232 10.1097/AJP.0000000000000975 PMC8500362

[ejp1988-bib-0020] Cipriani, A. , Furukawa, T. A. , Salanti, G. , Chaimani, A. , Atkinson, L. Z. , Ogawa, Y. , Leucht, S. , Ruhe, H. G. , Turner, E. H. , Higgins, J. P. T. , Egger, M. , Takeshima, N. , Hayasaka, Y. , Imai, H. , Shinohara, K. , Tajika, A. , Ioannidis, J. P. A. , & Geddes, J. R. (2018). Comparative efficacy and acceptability of 21 antidepressant drugs for the acute treatment of adults with major depressive disorder: A systematic review and network meta‐analysis. Lancet (London, England), 391, 1357–1366. 10.1016/S0140-6736(17)32802-7 PMC5889788 29477251

[ejp1988-bib-0021] De Tommaso, M. , Sardaro, M. , Pecoraro, C. , Di Fruscolo, O. , Serpino, C. , Lamberti, P. , & Livrea, P. (2007). Effects of the remote C fibres stimulation induced by capsaicin on the blink reflex in chronic migraine. Cephalalgia, 27, 881–890. 17593297 10.1111/j.1468-2982.2007.01357.x

[ejp1988-bib-0022] Edwards, R. R. , Cahalan, C. , Mensing, G. , Smith, M. , & Haythornthwaite, J. A. (2011). Pain, catastrophizing, and depression in the rheumatic diseases. Nature Reviews Rheumatology, 7, 216–224. 21283147 10.1038/nrrheum.2011.2

[ejp1988-bib-0023] Edwards, R. R. , Dolman, A. J. , Martel, M. O. , Finan, P. H. , Lazaridou, A. , Cornelius, M. , & Wasan, A. D. (2016). Variability in conditioned pain modulation predicts response to NSAID treatment in patients with knee osteoarthritis. BMC Musculoskeletal Disorders, 17, 284–292. 27412526 10.1186/s12891-016-1124-6 PMC4944243

[ejp1988-bib-0024] Escobar, A. , Quintana, J. M. , Bilbao, A. , Azkárate, J. , Güenaga, J. I. , Arenaza, J. C. , & Gutierrez, L. F. (2007). Effect of patient characteristics on reported outcomes after total knee replacement. Rheumatology (Oxford), 46, 112–119. 16735451 10.1093/rheumatology/kel184

[ejp1988-bib-0025] Forsythe, M. E. , Dunbar, M. J. , Hennigar, A. W. , Sullivan, M. J. L. , & Gross, M. (2008). Prospective relation between catastrophizing and residual pain following knee arthroplasty: Two‐year follow‐up. Pain Research & Management, 13, 335–341. 18719716 10.1155/2008/730951 PMC2671320

[ejp1988-bib-0026] Frakes, E. P. , Risser, R. C. , Ball, T. D. , Hochberg, M. C. , & Wohlreich, M. M. (2011). Duloxetine added to oral nonsteroidal anti‐inflammatory drugs for treatment of knee pain due to osteoarthritis: Results of a randomized, double‐blind, placebo‐controlled trial. Current Medical Research and Opinion, 27, 2361–2372. 22017192 10.1185/03007995.2011.633502

[ejp1988-bib-0027] Gewandter, J. S. , McDermott, M. P. , Evans, S. , Katz, N. P. , Markman, J. D. , Simon, L. S. , Turk, D. C. , & Dworkin, R. H. (2021). Composite outcomes for pain clinical trials: Considerations for design and interpretation. Pain, 162, 1899–1905. 33449513 10.1097/j.pain.0000000000002188 PMC8991304

[ejp1988-bib-0028] Graven‐Nielsen, T. , Izumi, M. , Petersen, K. K. , & Arendt‐Nielsen, L. (2017). User‐independent assessment of conditioning pain modulation by cuff pressure algometry. European Journal of Pain, 21, 552–561. 27859944 10.1002/ejp.958

[ejp1988-bib-0029] Graven‐Nielsen, T. , Wodehouse, T. , Langford, R. M. , Arendt‐Nielsen, L. , & Kidd, B. L. (2012). Normalization of widespread hyperesthesia and facilitated spatial summation of deep‐tissue pain in knee osteoarthritis patients after knee replacement. Arthritis and Rheumatism, 64, 2907–2916. 22421811 10.1002/art.34466

[ejp1988-bib-0030] Hansen, S. , Vaegter, H. B. , & Petersen, K. K. (2020). Pretreatment exercise‐induced hypoalgesia is associated with change in pain and function after standardized exercise therapy in painful knee osteoarthritis. The Clinical Journal of Pain, 36, 16–24. 31567220 10.1097/AJP.0000000000000771

[ejp1988-bib-0031] Heinze, G. , & Dunkler, D. (2017). Five myths about variable selection. Transplant International, 30, 6–10. 27896874 10.1111/tri.12895

[ejp1988-bib-0032] Heredia‐Rizo, A. M. , Petersen, K. K. , Madeleine, P. , & Arendt‐Nielsen, L. (2019). Clinical outcomes and central pain mechanisms are improved after upper trapezius eccentric training in female computer users with chronic neck/shoulder pain. The Clinical Journal of Pain, 35, 65–76. 30222615 10.1097/AJP.0000000000000656

[ejp1988-bib-0033] Holm, P. M. , Petersen, K. K. , Wernbom, M. , Schrøder, H. M. , Arendt‐Nielsen, L. , & Skou, S. T. (2021). Strength training in addition to neuromuscular exercise and education in individuals with knee osteoarthritis‐the effects on pain and sensitization. European Journal of Pain, 25, 1898–1911. 33991370 10.1002/ejp.1796

[ejp1988-bib-0034] Imai, Y. , Petersen, K. K. , Mørch, C. D. , & Arendt Nielsen, L. (2016). Comparing test–retest reliability and magnitude of conditioned pain modulation using different combinations of test and conditioning stimuli. Somatosensory & Motor Research, 33, 169–177. 27650216 10.1080/08990220.2016.1229178

[ejp1988-bib-0035] Izumi, M. , Petersen, K. K. , Laursen, M. B. , Arendt‐Nielsen, L. , & Graven‐Nielsen, T. (2017). Facilitated temporal summation of pain correlates with clinical pain intensity after hip arthroplasty. Pain, 158, 323–332. 27870648 10.1097/j.pain.0000000000000764

[ejp1988-bib-0036] Jin, Y. , Sun, T. , Zheng, P. , & An, J. (2021). Mass quarantine and mental health during COVID‐19: A meta‐analysis. Journal of Affective Disorders, 295, 1335–1346. 34706447 10.1016/j.jad.2021.08.067 PMC8674683

[ejp1988-bib-0037] Kendell, K. , Saxby, B. , Farrow, M. , & Naisby, C. (2001). Psychological factors associated with short‐term recovery from total knee replacement. British Journal of Health Psychology, 6, 41–52. 14596737 10.1348/135910701169043

[ejp1988-bib-0038] Kennedy, D. L. , Kemp, H. I. , Ridout, D. , Yarnitsky, D. , & Rice, A. S. (2016). Reliability of conditioned pain modulation. Pain, 157, 2410–2419. 27559835 10.1097/j.pain.0000000000000689 PMC5228613

[ejp1988-bib-0039] Kisler, L. B. , Weissman‐Fogel, I. , Coghill, R. C. , Sprecher, E. , Yarnitsky, D. , & Granovsky, Y. (2019). Individualization of migraine prevention. The Clinical Journal of Pain, 35, 753–765. 31241488 10.1097/AJP.0000000000000739

[ejp1988-bib-0040] Kjær, P. , Olesen, A. E. , Simonsen, O. , & Arendt‐Nielsen, L. (2019). Mechanistic pain profiling as a tool to predict the efficacy of 3‐week nonsteroidal anti‐inflammatory drugs plus paracetamol in patients with painful knee osteoarthritis. Pain, 160, 486–492. 30371559 10.1097/j.pain.0000000000001427

[ejp1988-bib-0041] Koh, I. J. , Kim, M. S. , Sohn, S. , Song, K. Y. , Choi, N. Y. , & In, Y. (2019). Duloxetine reduces pain and improves quality of recovery following total knee arthroplasty in centrally sensitized patients. Journal of Bone and Joint Surgery, 101, 64–73. 10.2106/JBJS.18.00347 30601417

[ejp1988-bib-0042] Kosek, E. , & Ordeberg, G. (2000). Lack of pressure pain modulation by heterotopic noxious conditioning stimulation in patients with painful osteoarthritis before, but not following, surgical pain relief. Pain, 88, 69–78. 11098101 10.1016/S0304-3959(00)00310-9

[ejp1988-bib-0043] Kristensen, N. S. , Hertel, E. , Skadhauge, C. H. , Kronborg, S. H. , Petersen, K. K. , & McPhee, M. E. (2021). Psychophysical predictors of experimental muscle pain intensity following fatiguing calf exercise. PLoS One, 16, e0253945. 34329324 10.1371/journal.pone.0253945 PMC8323909

[ejp1988-bib-0044] Kurien, T. , Arendt‐Nielsen, L. , Petersen, K. K. , Graven‐Nielsen, T. , & Scammell, B. E. (2018). Preoperative neuropathic pain‐like symptoms and central pain mechanisms in knee osteoarthritis predicts poor outcome 6 months after Total knee replacement surgery. The Journal of Pain, 19, 1329–1341. 29920331 10.1016/j.jpain.2018.05.011

[ejp1988-bib-0045] Larsen, D. B. , Laursen, M. , Edwards, R. R. , Simonsen, O. , Arendt‐Nielsen, L. , & Petersen, K. K. (2021). The combination of preoperative pain, conditioned pain modulation, and pain catastrophizing predicts postoperative pain 12 months after Total knee arthroplasty. Pain Medicine, 22, 1583–1590. 33411890 10.1093/pm/pnaa402

[ejp1988-bib-0046] Larsen, D. B. , Laursen, M. B. , Simonsen, O. H. , Arendt‐Nielsen, L. , & Petersen, K. K. (2021). The association between sleep quality, preoperative risk factors for chronic postoperative pain, and postoperative pain intensity 12 months after knee and hip arthroplasty. British Journal of Pain, 15, 486–496. 34840796 10.1177/20494637211005803 PMC8611299

[ejp1988-bib-0047] Lockwood, S. M. , Bannister, K. , & Dickenson, A. H. (2019). An investigation into the noradrenergic and serotonergic contributions of diffuse noxious inhibitory controls in a monoiodoacetate model of osteoarthritis. Journal of Neurophysiology, 121, 96–104. 30461363 10.1152/jn.00613.2018 PMC6383660

[ejp1988-bib-0048] Lunn, T. H. , Frokjaer, V. G. , Hansen, T. B. , Kristensen, P. W. , Lind, T. , & Kehlet, H. (2015). Analgesic effect of perioperative escitalopram in high pain catastrophizing patients after total knee arthroplasty: A randomized, double‐blind, placebo‐controlled trial. Anesthesiology, 122, 884–894. 25782644 10.1097/ALN.0000000000000597

[ejp1988-bib-0049] Momenimovahed, Z. , Salehiniya, H. , Hadavandsiri, F. , Allahqoli, L. , Günther, V. , & Alkatout, I. (2021). Psychological distress among cancer patients during COVID‐19 pandemic in the world: A systematic review. Frontiers in Psychology, 12, 682154. 34650469 10.3389/fpsyg.2021.682154 PMC8506116

[ejp1988-bib-0050] OʼLeary, H. , Smart, K. M. , Moloney, N. A. , Blake, C. , & Doody, C. M. (2018). Pain sensitization associated with nonresponse after physiotherapy in people with knee osteoarthritis. Pain, 159, 1877–1886. 29794610 10.1097/j.pain.0000000000001288

[ejp1988-bib-0051] Petersen, K.K. (2021). Mechanistic pain profiling of patients with osteoarthritis ‐ current knowledge and future directions (Dr.Med Thesis, AAU Press).

[ejp1988-bib-0052] Petersen, K. K. , Andersen, H. H. , Tsukamoto, M. , Tracy, L. , Koenig, J. , & Arendt‐Nielsen, L. (2018). The effects of propranolol on heart rate variability and quantitative, mechanistic, pain profiling: A randomized placebo‐controlled crossover study. Scandinavian Journal of Pain, 18(3), 479–489. 29858911 10.1515/sjpain-2018-0054

[ejp1988-bib-0053] Petersen, K. K. , Arendt‐Nielsen, L. , Simonsen, O. , Wilder‐Smith, O. , & Laursen, M. B. (2015). Presurgical assessment of temporal summation of pain predicts the development of chronic postoperative pain 12 months after total knee replacement. Pain, 156, 55–61. 25599301 10.1016/j.pain.0000000000000022

[ejp1988-bib-0054] Petersen, K. K. , Graven‐Nielsen, T. , Simonsen, O. , Laursen, M. B. M. B. , & Arendt‐Nielsen, L. (2016). Preoperative pain mechanisms assessed by cuff algometry are associated with chronic postoperative pain relief after total knee replacement. Pain, 157, 1400–1406. 27331347 10.1097/j.pain.0000000000000531

[ejp1988-bib-0055] Petersen, K. K. , Simonsen, O. , Laursen, M. B. , & Arendt‐Nielsen, L. (2018). The role of preoperative radiologic severity, sensory testing, and temporal summation on chronic postoperative pain following total knee arthroplasty. The Clinical Journal of Pain, 34, 193–197. 28654559 10.1097/AJP.0000000000000528

[ejp1988-bib-0056] Petersen, K. K. , Simonsen, O. , Olesen, A. E. , Mørch, C. D. , & Arendt‐Nielsen, L. (2019). Pain inhibitory mechanisms and response to weak analgesics in patients with knee osteoarthritis. European Journal of Pain, 23, 1904–1912. 31376308 10.1002/ejp.1465

[ejp1988-bib-0057] Petersen, K. K. , Vaegter, H. B. , Stubhaug, A. , Wolff, A. , Scammell, B. E. , Arendt‐Nielsen, L. , & Larsen, D. B. (2021). The predictive value of quantitative sensory testing: A systematic review on chronic postoperative pain and the analgesic effect of pharmacological therapies in patients with chronic pain. Pain, 162, 31–44. 32701654 10.1097/j.pain.0000000000002019

[ejp1988-bib-0058] Posner, K. , Brown, G. K. , Stanley, B. , Brent, D. A. , Yershova, K. V. , Oquendo, M. A. , Currier, G. W. , Melvin, G. A. , Greenhill, L. , Shen, S. , & Mann, J. J. (2011). The Columbia–suicide severity rating scale: Initial validity and internal consistency findings from three multisite studies with adolescents and adults. The American Journal of Psychiatry, 168, 1266–1277. 22193671 10.1176/appi.ajp.2011.10111704 PMC3893686

[ejp1988-bib-0059] Reyes, C. , Leyland, K. M. , Peat, G. , Cooper, C. , Arden, N. K. , & Prieto‐Alhambra, D. (2016). Association between overweight and obesity and risk of clinically diagnosed knee, hip, and hand osteoarthritis: A population‐based cohort study. Arthritis & Rhematology, 68, 1869–1875. 10.1002/art.39707 PMC4966641 27059260

[ejp1988-bib-0060] Rienstra, W. , Blikman, T. , Dijkstra, B. , Stewart, R. , Zijlstra, W. , van Raaij, T. , ten Hagen, A. , Bulstra, S. , Stevens, M. , & van den Akker‐Scheek, I. (2021). Effect of preoperative duloxetine treatment on postoperative chronic residual pain after total hip or knee arthroplasty: A randomised controlled trial. BMJ Open, 11, e052944. 10.1136/bmjopen-2021-052944 PMC8572398 34732491

[ejp1988-bib-0061] Safiri, S. , Kolahi, A.‐A. , Smith, E. , Hill, C. , Bettampadi, D. , Mansournia, M. A. , Hoy, D. , Ashrafi‐Asgarabad, A. , Sepidarkish, M. , Almasi‐Hashiani, A. , Collins, G. , Kaufman, J. , Qorbani, M. , Moradi‐Lakeh, M. , Woolf, A. D. , Guillemin, F. , March, L. , & Cross, M. (2020). Global, regional and national burden of osteoarthritis 1990‐2017: A systematic analysis of the global burden of disease study 2017. Annals of the Rheumatic Diseases, 79, 819–828. 32398285 10.1136/annrheumdis-2019-216515

[ejp1988-bib-0062] Staffe, A. T. , Bech, M. W. , Clemmensen, S. L. K. , Nielsen, H. T. , Larsen, D. B. , & Petersen, K. K. (2019). Total sleep deprivation increases pain sensitivity, impairs conditioned pain modulation and facilitates temporal summation of pain in healthy participants. PLoS One, 14, e0225849. 31800612 10.1371/journal.pone.0225849 PMC6892491

[ejp1988-bib-0063] Sullivan, M. J. L. , Bishop, S. R. , & Pivik, J. (1995). The pain catastrophizing scale: Development and validation. Psychological Assessment, 7, 524–532.

[ejp1988-bib-0064] Uchio, Y. , Enomoto, H. , Ishida, M. , Tsuji, T. , Ochiai, T. , & Konno, S. (2018). Safety and efficacy of duloxetine in Japanese patients with chronic knee pain due to osteoarthritis: An open‐label, long‐term, phase III extension study. Journal of Pain Research, 11, 1391–1403. 30104894 10.2147/JPR.S171395 PMC6074806

[ejp1988-bib-0065] Wang, G. , Bi, L. , Li, X. , Li, Z. , Zhao, D. , Chen, J. , He, D. , Wang, C.‐N. , Wu, T. , Dueñas, H. , Skljarevski, V. , & Yue, L. (2019). Maintenance of effect of duloxetine in Chinese patients with pain due to osteoarthritis: 13‐week open‐label extension data. BMC Musculoskeletal Disorders, 20, 174–182. 31010413 10.1186/s12891-019-2527-y PMC6477726

[ejp1988-bib-0066] Wang, Z. Y. , Shi, S. Y. , Li, S. J. , Chen, F. , Chen, H. , Lin, H. Z. , & Lin, J. M. (2015). Efficacy and safety of duloxetine on osteoarthritis knee pain: A meta‐analysis of randomized controlled trials. Pain Med (United States), 16, 1373–1385. 10.1111/pme.12800 26176791

[ejp1988-bib-0067] Yarnitsky, D. (2010). Conditioned pain modulation (the diffuse noxious inhibitory control‐like effect): Its relevance for acute and chronic pain states. Current Opinion in Anaesthesiology, 23, 611–615. 20543676 10.1097/ACO.0b013e32833c348b

[ejp1988-bib-0068] Yarnitsky, D. , Granot, M. , Nahman‐Averbuch, H. , Khamaisi, M. , & Granovsky, Y. (2012). Conditioned pain modulation predicts duloxetine efficacy in painful diabetic neuropathy. Pain, 153, 1193–1198. 22480803 10.1016/j.pain.2012.02.021

[ejp1988-bib-0069] Zigmond, A. , & Snaith, R. (1983). The hospital anxiety and depression scale. Acta Psychiatrica Scandinavica, 67, 361–370. 6880820 10.1111/j.1600-0447.1983.tb09716.x

