# Supplementary material for: The effect of duloxetine on mechanistic pain profiles, cognitive factors and clinical pain in patients with painful knee osteoarthritis—A randomized, double‐blind, placebo‐controlled, crossover study
Source: Eur J Pain. 2022 Jun 13;26(8):1650–64. doi: 10.1002/ejp.1988 (PMC9541875; doi:10.1002/ejp.1988)
Supplement: Supplementary file 1 — Table S1 [file EJP-26-1650-s001.docx]

## Supplementary table 1

| Model 1 | Standardized β-values | | | |
| --- | --- | --- | --- | --- |
|  | BPI worst pain | BPI average pain | WOMAC pain | WOMAC total |
| Adjusted R^2^ | 5.9% | 12.2% | 20.5% | 4.8% |
|  |  |  |  |  |
| cPDT (ipsi) | 0.570 | 0.259 | 0.275 | 0.162 |
| cPDT (contra) | 0.329 | 0.208 | 0.124 | 0.132 |
| cPTT (ipsi) | 0.185 | 0.367 | 0.409 | 0.320 |
| cPTT (contra) | -0.786 | -0.500 | -0.579 | -0.656 |
| TSP | 0.272 | 0.424 | 0.423 | 0.205 |
| CPM | 0.319 | 0.202 | 0.121 | 0.129 |
| BPI Worst pain | 0.484 | -0.117 | -0.324 | 0.092 |
| BPI Average pain | -0.010 | 0.794 | 0.093 | -0.297 |
| WOMAC pain | 0.576 | 0.430 | 1.319 | 0.830 |
| WOMAC total | -0.660 | -0.690 | -1.048 | -0.392 |
| HADS | -0.141 | -0.173 | 0.048 | -0.036 |
| PCS | 0.221 | 0.342 | 0.472 | 0.358 |
|  |  |  |  |  |
| Model 2 | Standardized β-values | | | |
|  | Worst pain | Average pain | WOMAC pain | WOMAC total |
| Adjusted R^2^ | 22.0% | 32.0% | 41.8% | 28.1% |
|  |  |  |  |  |
| cPDT (ipsi) |  |  |  |  |
| cPDT (contra) |  |  |  |  |
| cPTT (ipsi) |  |  |  |  |
| cPTT (contra) |  |  |  |  |
| TSP |  |  | 0.269 |  |
| CPM |  |  |  |  |
| BPI Worst pain | **0.505** |  |  |  |
| BPI Average pain |  | **0.593** |  |  |
| WOMAC pain |  |  | **1.315** | **0.560** |
| WOMAC total |  |  | **-0.909** |  |
| HADS |  |  |  |  |
| PCS |  |  |  |  |

**Supplementary table 1:** Multiple linear regression models aiming to establish the adjusted predictive value (**R^2^**) for baseline parameters predicting analgesic response to placebo treatment. Model 1 contains all baseline parameters, whereas Model 2 was constructed using backwards selection and aimed to identify independent predictors (bold numbers in model 2 are significant independent predictors). **Abbreviations**: **ipsi**: ipsilateral side to the most osteoarthritic affected knee, **contra**: contralateral side to the most osteoarthritic affected knee, **TSP**: temporal summation of pain, **CPM**: conditioned pain modulation, **BPI**: Brief Pain Inventory, **WOMAC**: Western Ontario and McMaster Universities Osteoarthritis Index, **HADS**: Hospital Anxiety and Depression Score, **PCS**: Pain Catastrophizing Scale, **cPDT**: cuff pain detection threshold, **cPTT**: cuff pressure pain tolerance threshold.
